# Tobacco smoke exposure and spine fracture risk in US adults: A cross-sectional analysis of the National Health and Nutrition Examination Survey 1999–2010, 2013–2014, 2017–2020

**DOI:** 10.18332/tid/217959

**Published:** 2026-06-08

**Authors:** Rui Zhang, Wei Long, Yanchao Li

**Affiliations:** 1 Department of Orthopedics, The First People’s Hospital of Yibin, Yibin, China; 2 Department of Ophthalmology, The First People’s Hospital of Yibin, Yibin, China; 3 School of Graduates, Dalian Medical University, Dalian, China

**Keywords:** tobacco smoke exposure, spine fracture, serum cotinine, linearity, NHANES

## Abstract

**INTRODUCTION:**

While tobacco smoke is an established risk factor for multiple pathological conditions, its specific relationship with spine fracture remains inadequately characterized in the scientific literature. This study was designed to assess the potential link between tobacco smoke exposure and spine fracture prevalence among US adults, and to investigate the presence of a dose-response gradient.

**METHODS:**

We performed an analytical cross-sectional study utilizing data from the National Health and Nutrition Examination Survey (NHANES) 1999–2010, 2013–2014, 2017–2020. Serum cotinine concentrations were employed as an objective biomarker for tobacco smoke exposure. Multivariable logistic regression models were constructed to quantify the association between smoke exposure and a diagnosis of spine fracture. We performed a rigorous evaluation of the association's consistency through subgroup stratification. Further, we characterized the dose-response relationship using restricted cubic splines (RCS) with threshold effect analysis to detect possible non-linear patterns.

**RESULTS:**

Our final analytical cohort included 31124 eligible participants. After comprehensive covariate adjustment, multivariable analysis revealed a significant positive correlation between tobacco smoke exposure and spine fracture risk. When treated as a continuous variable, a significant positive linear dose-response relationship was observed: the natural log-transformed cotinine concentration maintained a significant positive association with spine fracture risk across all progressively adjusted models (OR=1.04; 95% CI: 1.02–1.06). As a categorical variable, participants with serum cotinine concentrations indicative of active smoking (≥3 ng/mL) demonstrated a 41% elevated spine fracture risk compared to those with unexposed participants (<0.05 ng/mL, reference group). The consistency of the observed association was further affirmed through stratified analyses and interaction testing across all demographic and clinical subgroups, lending robust support to our primary finding.

**CONCLUSIONS:**

Our analysis provides evidence that tobacco smoke exposure is independently associated with an increased risk of spine fracture. These findings underscore the public health imperative to implement tobacco control measures.

## INTRODUCTION

Spine fracture represents a substantial global health burden, contributing significantly to disability, chronic pain management challenges, functional impairment, and substantial healthcare expenditures^1,2^. Epidemiological data indicate a rising global incidence of these fractures. For instance, Finnish population registries documented a 57% increase in spine fracture hospitalization rates between 1998 and 2017, rising from 57 to 89 cases per 100000 person^3^. Similarly, German health statistics reported a 45.6% surge in spine fracture incidence from 2009 to 2019, reaching 150.7 cases per 100000 population^4^. The significant morbidity and economic impact associated with these injuries underscore the critical need for effective preventive strategies and early detection methods.

Tobacco consumption remains a leading preventable cause of global mortality and morbidity. Despite substantial progress in tobacco control initiatives, approximately 1.3 billion individuals worldwide continue to use tobacco products, resulting in over 7 million annual deaths attributable to tobacco-related diseases^5^. In developed nations, the mortality burden primarily manifests through malignancies (lung, upper digestive tract, bladder), chronic obstructive pulmonary disease, and cardiovascular disorders^6^. Both direct smoking and secondhand smoke inhalation have demonstrated detrimental effects on skeletal health. Mechanistic studies indicate that tobacco smoke disrupts normal bone remodeling cycles, promoting accelerated bone loss and osteoporosis development, thereby increasing fracture susceptibility^7^. Beyond initial fracture risk, tobacco users experience compromised bone healing capacity and delayed fracture union, with some skeletal impairments persisting for years following cessation^8^. Current epidemiological evidence specifically addressing the tobacco-spine fracture relationship remains limited and inconclusive. Cotinine, the primary nicotine metabolite with superior pharmacokinetic properties, including an extended half-life and consistent metabolic clearance, provides a highly reliable exposure biomarker. Consequently, serum cotinine measurement represents the gold standard for quantifying tobacco smoke exposure in biomedical research^9^.

The present study aims to comprehensively evaluate the association between biologically quantified tobacco smoke exposure and spine fracture prevalence through a secondary analysis of cross-sectional data from the National Health and Nutrition Examination Survey (NHANES) 1999–2010, 2013–2014, 2017–2020. By extending these epidemiological observations, we seek to enhance understanding of tobacco’s skeletal effects and inform evidence-based fracture prevention strategies.

## METHODS

### Data sources and study population

**Figure 1:**
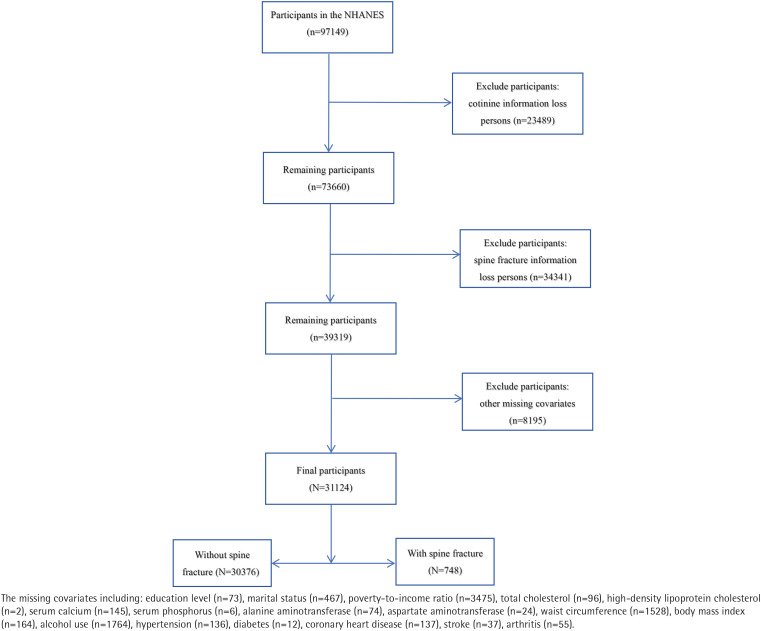
Study flow diagram for the cross-sectional analysis of smoking and spine fracture in the US, NHANES 1999–2010, 2013–2014, 2017–2020 (N=31124)

The analysis drew on nationally representative data from the National Health and Nutrition Examination Survey (NHANES)^10^, a comprehensive study conducted by the National Center for Health Statistics (NCHS) that evaluates the health and nutritional status of children and adults in the United States. A multistage, probability-based sampling strategy is employed in the survey to ensure that the derived estimates accurately reflect the target population^10^. The NHANES survey protocol was reviewed and approved by the National Center for Health Statistics (NCHS) Institutional Review Board. All participants provided written informed consent. As the present study constitutes a secondary analysis of de-identified, publicly available data, it was exempt from additional ethical review by an institutional review board. This research was conducted in accordance with the ethical principles outlined in the Declaration of Helsinki, while reporting standards followed the STROBE checklist for cross-sectional studies. We aggregated data from eight survey cycles (1999–2000, 2001–2002, 2003–2004, 2005–2006, 2007–2008, 2009–2010, 2013–2014, 2017–2020), initially identifying 97149 potential participants aged ≥20 years. After sequentially excluding individuals with missing cotinine measurements (n=23489), undetermined fracture status (n=34341), and incomplete covariate data (n=8195), our final analytical sample comprised 31124 participants (Figure 1).

### Assessment of tobacco smoke exposure

Tobacco smoke exposure was assessed by measuring serum cotinine, which serves as a proxy for both direct inhalation and passive inhalation of environmental tobacco smoke^11^. The analytical method involved isotope-dilution high-performance liquid chromatography/atmospheric pressure chemical ionization tandem mass spectrometry (ID HPLC-APCI MS/MS). The analytical capacity of this method includes a lower limit of detection of 0.015 ng/mL and an intra-assay coefficient of variation (CV) of <10%. These technical specifications ensure high precision and reliability in quantifying serum cotinine levels. For categorical analysis, we adopted the cut-point recommended by Benowitz et al.^12^ to define exposure levels: concentrations <0.05 ng/mL were classified as ‘Unexposed’; 0.05–2.99 ng/mL indicated ‘Low exposure’ from passive smoking; and levels ≥3 ng/mL identified ‘Heavy exposure’ consistent with active smoking.

### Spine fracture definition

Spine fracture was operationalized as a self-reported, physician-diagnosed event, based on the following NHANES interview question from the Medical Conditions section: ‘Has a doctor or other health professional ever told you that you had a fracture or broken bone in your spine?’. Participants who responded ‘Yes’ were classified as having a spine fracture; those responding ‘No’ or ‘Don’t know’ were classified as non-cases. This epidemiological criterion for case identification has demonstrated adequate authenticity and is considered a feasible method in large-scale population studies, consistent with previous findings^13^.

### Covariables data extraction

The NHANES protocol involved two primary data-collection modalities: standardized household interviews and comprehensive physical examinations conducted in mobile examination centers. Covariates for this analysis were selected *a priori* based on their established relevance in the scientific literature and clinical rationale. The set of adjustment variables encompassed demographic factors (age, gender, marital status, race/ethnicity, education level, family income), existing medical conditions (hypertension, diabetes, coronary heart disease [CHD], stroke, arthritis), anthropometric measurements (waist circumference [WC], body mass index [BMI]), behavioral factors (alcohol use), and a panel of serum biomarkers (total cholesterol [TC, mmol/L], high-density lipoprotein cholesterol [HDL-C, mmol/L], calcium [mg/dL], phosphorus [mg/dL], alanine aminotransferase [ALT, U/L], aspartate aminotransferase [AST, U/L], blood urea nitrogen [BUN, mg/dL], and uric acid [UA, mg/dL]). Marital status was dichotomized as married/cohabitating or living alone. Self-reported race/ethnicity was categorized as Non-Hispanic White, Non-Hispanic Black, Mexican American, or Other. Education level was divided into three groups: <9, 9–12, and >12 years. Family income level was determined using the poverty income ratio (PIR) and classified as low (PIR ≤ 1.3), medium (1.3< PIR ≤ 3.5), or high (PIR >3.5), consistent with federal guidelines^14^. The presence of comorbidities (hypertension, diabetes, CHD, stroke, arthritis) was ascertained via self-report of a prior physician diagnosis. Waist circumference was measured at the iliac crest using a standardized protocol. BMI was calculated as weight (kg) divided by height (m) squared and categorized as: underweight/normal <25, overweight 25–29.9, and obese ≥30 kg/m^2^ according to WHO criteria. Alcohol use was defined as the consumption of at least 12 alcoholic drinks in the past year, based on the NHANES Alcohol Use Questionnaire.

### Weights processing

To account for the complex, multistage probability sampling design of NHANES and to produce nationally representative estimates, appropriate sample weights were applied in accordance with the NHANES analytical guidelines. Specifically, the weight variables used were WTMEC4YR (for 1999–2000 and 2001–2002), WTMEC2YR (for 2003–2004, 2005–2006, 2007–2008, 2009–2010, 2013–2014), and WTMECPRP (for 2017–2020). To create a single, analyzable weight for the combined dataset spanning all cycles, we followed the standard procedure recommended by the National Center for Health Statistics (NCHS). The weighting methodology proceeded as follows: initial standardization of raw weights within each survey period, followed by merging of standardized weights across multiple cycles to create a composite weight, which was then incorporated into all analytical models to produce estimates generalizable to the non-institutionalized US population^15^.

### Statistical analysis

Our secondary analysis of publicly available data employed distribution-appropriate statistical approaches. Continuous variables with normal distributions are reported as means ± SD; non-normal variables as medians (IQR); and categorical variables as frequencies and percentages. Intergroup comparisons utilized ANOVA (normal), Kruskal-Wallis (non-normal), or chi-squared (categorical) tests. The distribution of serum cotinine violated normality assumptions, necessitating a natural log transformation prior to treating it as a continuous variable in sensitivity analyses.

To quantify the relationship between tobacco smoke exposure and spine fracture risk, we computed odds ratios (ORs) with corresponding 95% confidence intervals (95% CIs) through multivariable logistic regression. We constructed four sequential models with progressive adjustment: Model 1 constituted a crude, unadjusted analysis; Model 2 incorporated sociodemographic confounders (age, gender, race, marital status, education level, and family income); Model 3 further adjusted for anthropometric and biochemical parameters (WC, BMI, TC, HDL-C, calcium, phosphorus, ALT, AST, BUN, UA); and Model 4 represented the fully adjusted model, additionally controlling for behavioral and clinical comorbidities (alcohol use, hypertension, diabetes, CHD, stroke, arthritis). The goodness-of-fit of the final multivariable logistic regression model was evaluated using the Hosmer-Lemeshow test. After adjusting for the covariates specified in Model 4, in the restricted cubic spline (RCS) analysis, we used 4 knots, a common choice that provides sufficient flexibility without overfitting in large datasets. The knots were placed at the 5th, 35th, 65th, and 95th percentiles of the natural log-transformed serum cotinine distribution, following standard recommendations for percentile-based knot placement to ensure stable estimates across the exposure range. The assessment of linearity versus non-linearity was conducted formally using the Wald test. This test evaluates the null hypothesis that the coefficients for all nonlinear spline terms are simultaneously equal to zero. A non-significant p-value supports a linear relationship. Furthermore, we also conducted extensive subgroup analyses. The subgroups used for stratification and interaction testing were predefined based on their established clinical relevance and potential as effect modifiers in previous literature. These included age (<40 vs ≥40 years), gender, race, marital status (married/partnered vs living alone), poverty-income ratio, alcohol use, hypertension, diabetes, CHD, stroke, and arthritis.

All data analyses were performed using R (version 4.2.1; The R Foundation) and Free Statistics software platforms (version 2.1.1). The study population characteristics were summarized using descriptive statistics. A two-tailed significance threshold of p<0.05 was employed for all inferential statistical tests.

## RESULTS

### Basic characteristics

Table 1 summarizes the baseline demographic and clinical characteristics of the 31124 participants in the final analysis, including 748 spine fracture cases (2.4%). Demographic data show a mean age of 52.7 years and 49.1% male representation. Notably, significant disparities were discernible across all measured covariates, spanning demographic, anthropometric, biochemical, and clinical domains, when comparing the different exposure categories.

**Table 1:** Baseline characteristics of participants in the cross-sectional study of tobacco smoke exposure and spine fracture in the US, NHANES 1999–2010 2013–2014, 2017–2020 (N=31124)

*Characteristics*	*Total* *(N=31124)*	*Unexposed* *(N=15618)*	*Low exposure* *(N=7501)*	*Heavy exposure* *(N=8005)*	*p*
**Age** (years)	52.7 ± 17.6	56.3 ± 17.2	50.7 ± 18.3	47.6 ± 16.1	<0.001
**Gender**					<0.001
Male	15271 (49.1)	6690 (42.8)	3770 (50.3)	4811 (60.1)	
Female	15853 (50.9)	8928 (57.2)	3731 (49.7)	3194 (39.9)	
**Race**					<0.001
Non-Hispanic White	15403 (49.5)	7853 (50.3)	3342 (44.6)	4208 (52.6)	
Non-Hispanic Black	6062 (19.5)	2037 (13)	1988 (26.5)	2037 (25.4)	
Mexican American	5452 (17.5)	3288 (21.1)	1236 (16.5)	928 (11.6)	
Other	4207 (13.5)	2440 (15.6)	935 (12.5)	832 (10.4)	
**Education level** (years)					<0.001
<9	3530 (11.3)	1855 (11.9)	887 (11.8)	788 (9.8)	
9–12	12059 (38.7)	4682 (30)	3206 (42.7)	4171 (52.1)	
>12	15535 (49.9)	9081 (58.1)	3408 (45.4)	3046 (38.1)	
**Marital status**					<0.001
Married/living with partner	19381 (62.3)	10782 (69)	4357 (58.1)	4242 (53)	
Living alone	11743 (37.7)	4836 (31)	3144 (41.9)	3763 (47)	
**PIR**					<0.001
Low	8695 (27.9)	3154 (20.2)	2362 (31.5)	3179 (39.7)	
Medium	12040 (38.7)	6052 (38.8)	2926 (39)	3062 (38.3)	
High	10389 (33.4)	6412 (41.1)	2213 (29.5)	1764 (22)	
**Clinical status**					
TC (mmol/L)	5.1 ± 1.1	5.1 ± 1.1	5.1 ± 1.1	5.1 ± 1.1	<0.001
HDL-C (mmol/L)	1.4 ± 0.4	1.4 ± 0.4	1.4 ± 0.4	1.3 ± 0.4	<0.001
Serum calcium (mg/dL)	9.4 ± 0.4	9.4 ± 0.4	9.4 ± 0.4	9.5 ± 0.4	<0.001
Serum phosphorus (mg/dL)	3.7 ± 0.6	3.7 ± 0.5	3.7 ± 0.6	3.7 ± 0.6	<0.001
ALT (U/L)	21.0 (16.0–28.0)	20.0 (16.0–27.0)	21.0 (16.0–29.0)	20.0 (16.0–28.0)	
AST (U/L)	22.0 (19.0–27.0)	22.0 (19.0–27.0)	23.0 (19.0–27.0)	22.0 (19.0–27.0)	
BUN (mg/dL)	14.0 ± 6.2	14.8 ± 6.3	14.1 ± 6.5	12.5 ± 5.6	<0.001
UA (mg/dL)	5.4 ± 1.5	5.3 ± 1.4	5.6 ± 1.5	5.5 ± 1.4	<0.001
WC (cm)	99.5 ± 15.7	99.5 ± 15.1	101.0 ± 16.3	98.0 ± 16.2	
BMI (kg/m^2^)	29.0 ± 6.5	29.0 ± 6.1	29.9 ± 7.0	28.0 ± 6.5	<0.001
**Alcohol use**					<0.001
No	8063 (25.9)	4714 (30.2)	2219 (29.6)	1130 (14.1)	
Yes	23061 (74.1)	10904 (69.8)	5282 (70.4)	6875 (85.9)	
**Hypertension**					<0.001
No	19292 (62.0)	9319 (59.7)	4624 (61.6)	5349 (66.8)	
Yes	11832 (38.0)	6299 (40.3)	2877 (38.4)	2656 (33.2)	
**Diabetes**					<0.001
No	26987 (86.7)	13354 (85.5)	6506 (86.7)	7127 (89)	
Yes	4137 (13.3)	2264 (14.5)	995 (13.3)	878 (11)	
**CHD**					<0.001
No	29545 (94.9)	14758 (94.5)	7128 (95)	7659 (95.7)	
Yes	1579 (5.1)	860 (5.5)	373 (5)	346 (4.3)	
**Stroke**					0.034
No	29840 (95.9)	15018 (96.2)	7178 (95.7)	7644 (95.5)	
Yes	1284 (4.1)	600 (3.8)	323 (4.3)	361 (4.5)	
**Arthritis**					<0.001
No	21587 (69.4)	10472 (67.1)	5392 (71.9)	5723 (71.5)	
Yes	9537 (30.6)	5146 (32.9)	2109 (28.1)	2282 (28.5)	
**Spine fracture**					<0.001
No	30376 (97.6)	15275 (97.8)	7345 (97.9)	7756 (96.9)	
Yes	748 (2.4)	343 (2.2)	156 (2.1)	249 (3.1)	

All statistical analyses in Table 1 were weighted using sample weights provided by NHANES to ensure that the results are nationally representative. Continuous variables with normal distributions are reported as means ± SD; non-normal variables as medians (IQR); and categorical variables as frequencies and percentages. P-values were derived from ANOVA (normal) Kruskal-Wallis (non-normal) or chi-squared (categorical) tests for intergroup comparisons. PIR: poverty-to-income ratio. TC: total cholesterol. HDL-C: high-density lipoprotein cholesterol. ALT: alanine aminotransferase. AST: aspartate aminotransferase. BUN: blood urea nitrogen. UA: uric acid. WC: waist circumference. BMI: body mass index. CHD: coronary heart disease.

### Univariable and multivariable logistic regression analyses

Preliminary univariable screening identified numerous variables significantly associated with spine fracture risk, including demographic factors, biochemical markers, and comorbidities (Supplementary file Table 1).

When treated as a continuous variable, univariable models showed that every unit increase in LN-transformed serum cotinine was associated with 5% higher odds of spine fracture (OR=1.05; 95% CI: 1.03–1.06). Table 2 presents the results of the multivariable logistic regression analysis examining the relationship between serum cotinine levels and spine fracture prevalence. After progressive adjustment (Model 4), the association remained robust: each unit increase in LN-cotinine corresponded to a 4% elevation in the odds of spine fracture (AOR=1.04; 95% CI: 1.02–1.06). We also performed a categorical analysis using the unexposed group (<0.05 ng/mL) as reference, which revealed a consistently elevated risk in the heavily exposed group (≥3 ng/mL). In contrast, the low exposure group (0.05–2.99 ng/mL) showed no significant association in any of the multivariable models (AOR=1.07; 95% CI: 0.87–1.30). The association for the heavy exposure group remained significant throughout the adjustment process, demonstrating odds ratios of 1.43 (95% CI: 1.21–1.69) in the crude model, 1.62 (95% CI: 1.35–1.95) after demographic adjustment, 1.60 (95% CI: 1.33–1.93) with additional biochemical/anthropometric adjustments, and 1.41 (95% CI: 1.16–1.70) in the fully adjusted model (Table 2).

**Table 2:** Multivariable logistic regression analysis the association between tobacco smoke exposure and spine fracture across different exposure levels in the US, NHANES 1999–2010, 2013–2014, 2017–2020 (N=31124)

*Variables*	*Model 1*		*Model 2*		*Model 3*		*Model 4*	
	*OR (95%CI)*	*p*	*AOR (95% CI)*	*p*	*AOR (95% CI)*	*p*	*AOR (95% CI)*	*p*
**LN-cotinine (ng/mL)**	1.05 (1.03–1.06)	<0.001	1.06 (1.04–1.08)	<0.001	1.06 (1.04–1.08)	<0.001	1.04 (1.02–1.06)	<0.001
**Tobacco smoke exposure**								
None (ref.)	1		1		1		1	
Low	0.95 (0.78–1.15)	0.568	1.09 (0.89–1.33)	0.395	1.07 (0.88–1.31)	0.498	1.07 (0.87–1.3)	0.521
Heavy	1.43 (1.21–1.69)	<0.001	1.62 (1.35–1.95)	<0.001	1.6 (1.33–1.93)	<0.001	1.41 (1.16–1.7)	<0.001
**Trend test**		<0.001		<0.001		<0.001		0.001

LN: natural logarithm. Model 1: unadjusted. AOR: adjusted odds ratio. Model 2: adjusted for age, gender, race, education level, marital status, PIR. Model 3: adjusted as in Model 2 plus TC, HDL-C, serum calcium, serum phosphorus, ALT, AST, BUN, UA, WC, BMI. Model 4: adjusted as in Model 3 plus alcohol use, hypertension, diabetes, CHD, stroke, arthritis.

### Model fit assessment

The goodness-of-fit of the multivariable logistic regression model was assessed using the Hosmer-Lemeshow test. The results indicated that the model fit the data well, with a non-significant chi-squared value (χ^2^=4.091, df=8) (Table 3).

**Table 3:** Hosmer-Lemeshow goodness-of-fit test for the multivariable logistic regression model of tobacco smoke exposure and spine fracture in the US, NHANES 1999–2010, 2013–2014, 2017–2020 (N=31124)

*Step*	*Chi-squared*	*df*	*Sig.*
1	4.091	8	0.849

### Restricted cubic spline models

**Figure 2:**
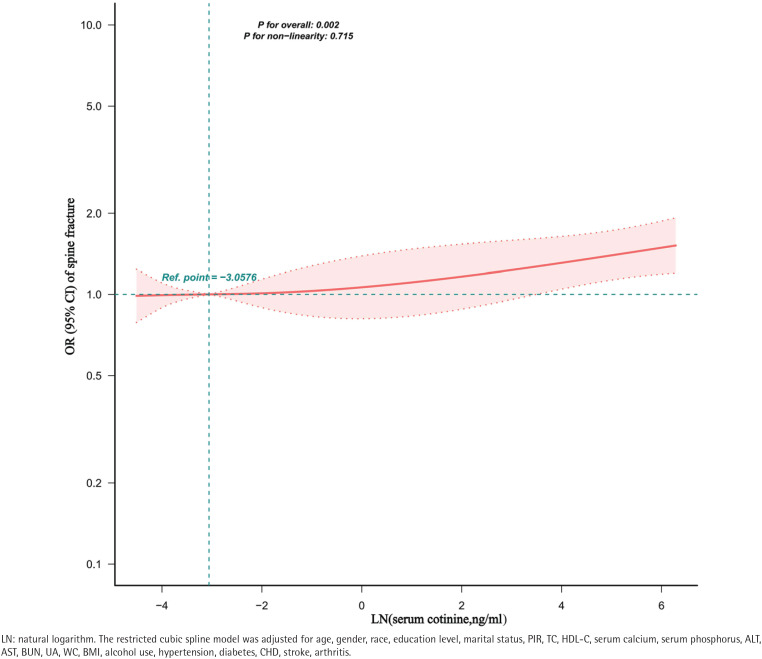
Restricted cubic spline analysis of the relationship between tobacco smoke exposure and spine fracture prevalence in the US, NHANES 1999–2010, 2013–2014, 2017–2020 (N=31124)

The restricted cubic spline analysis revealed that the relationship between LN-cotinine and spine fracture prevalence was effectively linear, as indicated by a non-significant test for non-linearity. These results are consistent with a linear dose-response pattern without evident threshold effects (Figure 2).

### Subgroup analysis

**Figure 3:**
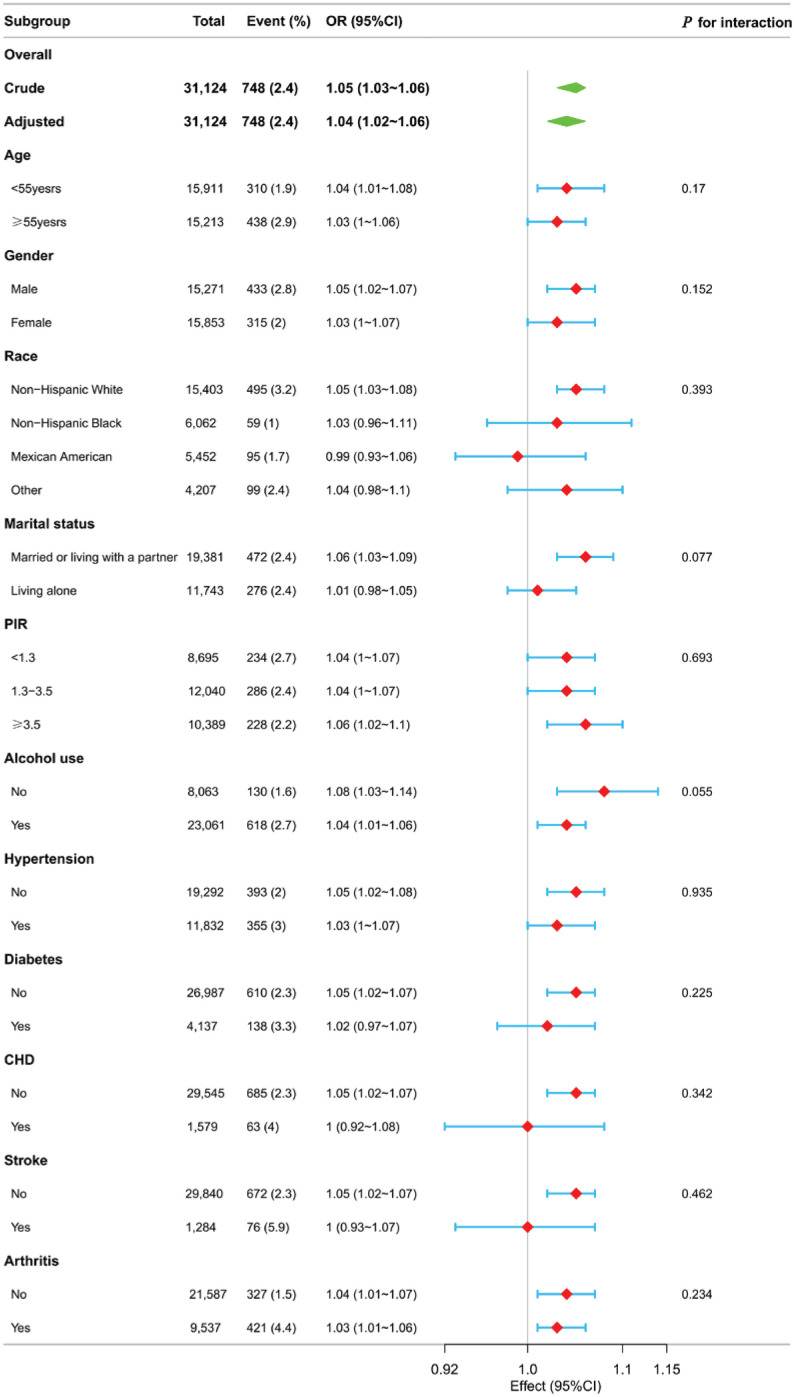
Forest plot of the association between tobacco smoke exposure and spine fracture prevalence across subgroups in the US, NHANES 1999–2010, 2013–2014, 2017–2020 (N=31124)

To evaluate the stability of the principal association, we performed extensive subgroup analyses stratified by age, gender, race, marital status, family income, alcohol use, and comorbidity status. Interaction testing revealed no significant effect modification by any of these stratification factors. The consistency of these results is visually presented in the forest plot (Figure 3) and detailed in Supplementary file Table 2.

## DISCUSSION

In this large, nationally representative cross-sectional study of US adults, we identified a significant linear relationship between objectively measured tobacco smoke exposure and spine fracture risk after comprehensive adjustment for potential confounders. The data revealed a pattern in which higher serum cotinine concentrations were associated with progressively higher spine fracture prevalence, suggesting a dose-response relationship. Our modeling strategy, which involved four sequential levels of covariate adjustment, consistently demonstrated this positive association. Most notably, the fully adjusted model (Model 4) established a significant 41% increase in fracture odds for individuals in the highest exposure category (serum cotinine ≥3 ng/mL) compared to the unexposed group. This linear dose-response relationship was further corroborated by restricted cubic spline analysis, which revealed no evidence of threshold effects or non-linearity. Furthermore, the absence of significant effect modification in our extensive subgroup analyses suggests that the observed association is consistent across diverse demographic and clinical subgroups, reinforcing its robustness as a population-level risk factor. Collectively, these findings indicate that heavy tobacco smoke exposure, as quantified by serum cotinine, is substantially associated with a higher prevalence of spine fracture.

Previous research into the relationship between tobacco smoke exposure and spine fracture has been sparse. Prior investigations have discovered that smoking is connected to a significantly increased risk of fractures^16,17^. Specifically, in men, smoking boosts the risk of spine fracture to 32% and hip fracture to 40%^18^. Ampelas^19^ reviewed studies investigating the connection between smoking and hip fracture. The results showed that the incidence of hip fracture was elevated in current smokers^19^. Substantial biological evidence confirms that tobacco smoke disrupts bone remodeling homeostasis, predisposing individuals to reduced bone mineral density and clinical osteoporosis, which consequently elevates fracture susceptibility^7^. Beyond this elevated risk, tobacco users frequently encounter compromised bone healing capacity, with some skeletal impairments persisting long after cessation due to the prolonged nature of these pathological effects^8^. Critically, previous epidemiological investigations in this domain have been constrained by non-standardized smoking definitions and limited generalizability due to non-representative sampling. Our investigation addresses several key methodological limitations in the existing literature. It is noteworthy, however, that some prior studies have reported non-significant associations between smoking and fracture risk^20,21^. These discrepancies may be attributed to several methodological factors. First, many earlier studies relied on self-reported smoking status, which is susceptible to recall and social desirability bias, potentially leading to exposure misclassification and an underestimation of the true effect. Second, differences in study population characteristics (e.g. age, sex distribution, ethnicity, osteoporosis prevalence) and the extent of adjustment for key confounders (such as bone mineral density, physical activity, or corticosteroid use) may also contribute to heterogeneous findings. The present study, by utilizing an objective biomarker of tobacco exposure and a large, nationally representative sample with comprehensive covariate adjustment, helps to mitigate these limitations and provides a more robust estimate of the association. This study partially overcomes these limitations by utilizing objective biomarkers and nationally representative data. While our findings contribute to this field, further validation through trials is warranted to establish causality and elucidate the underlying biological mechanisms.

The elucidation of smoking’s role in fracture etiology remains incomplete, though research has identified several putative pathways that may mediate this relationship. One plausible mechanism linking smoking to fracture is the decline in bone mineral density (BMD) resulting from smoking^22^. Having a low BMD is the chief cause of the risk associated with osteoporotic fractures. It serves as a broadly applied indicator in clinical practice for recognizing patients who are more likely to sustain fractures^23^. Jing et al.^24^ have implicated ferroptosis activation as a critical pathway in tobacco toxin-induced osteoporosis, demonstrating that this process in bone marrow mesenchymal stem cells leads to impaired bone formation, characterized by accumulation of intracellular reactive oxygen species that activate AMPK signaling. In addition, Li et al.^25^ found, based on the Swedish Osteoporotic Fracture cohort study, that cadmium from smoking partly contributes to the risk of osteoporosis. Another possible mechanism is that smoking leads to a decrease in calcium absorption^26^, and changes in vitamin D metabolism result in a decrease in vitamin D levels in the blood^27^, leading to an increased risk of fracture. While future studies must clarify the causal nature of this association, our results nevertheless illuminate a significant and modifiable risk factor with substantial clinical relevance. Given the observed association between tobacco exposure and spine fracture, targeted interventions to reduce smoking and environmental nicotine exposure are essential. Based on the results of this study, it is evident that minimizing active smoking may be a crucial factor in addressing a modifiable factor associated with spine fracture.

### Strengths and limitations

The nationwide and multi-ethnic scope of our study, based on the NHANES database with its complex sampling design, enhances the applicability and generalizability of our findings to the non-institutionalized US adult population. Furthermore, the large sample size (N=31124) provided adequate statistical power to detect the association of interest. In addition, a key methodological strength is the use of serum cotinine, an objective and quantitative biomarker, to assess tobacco smoke exposure. To our knowledge, this represents the first large-scale, population-based study to examine the specific relationship between tobacco smoke exposure (objectively measured by serum cotinine) and spine fracture risk. This approach minimizes misclassification bias inherent in self-reported smoking data and strengthens the internal validity of our exposure-outcome association. Finally, the application of complex survey weights in all analyses ensured that our estimates are representative of the non-institutionalized US adult population, further bolstering the external validity of our results.

However, several limitations should be noted. Firstly, the generalizability of our results may be constrained by the exclusive focus on the US population; external validation in diverse international cohorts is necessary. Secondly, the inherent constraints of the cross-sectional design preclude definitive causal inference regarding the relationship between tobacco smoke exposure and spine fracture. Thirdly, the assessment of serum cotinine at a single timepoint may not accurately reflect long-term exposure patterns, as it cannot account for intra-individual variability over time. Fourth, despite adjusting for a comprehensive set of covariates, residual confounding from unmeasured or imperfectly measured variables remains possible. Specifically, we lacked data on important potential confounders such as physical activity levels, dietary calcium and vitamin D intake, bone mineral density (BMD), history of falls, and use of medications affecting bone metabolism (e.g. glucocorticoids, bisphosphonates). Previous studies have identified these factors as significant predictors of fracture risk and may also be associated with smoking behavior, thus potentially confounding the observed association^28^. Fifth, the use of self-reported data for several variables, including spine fracture status and comorbidities, introduces the potential for recall bias and outcome misclassification. Finally, the exclusion of participants with missing data on cotinine, fracture status, or covariates (n=66025 excluded) may have introduced selection bias if these individuals differed systematically from those included in the analytical sample in terms of both exposure and outcome. Nevertheless, the large sample size and use of survey weights aim to mitigate the impact of this limitation on population-level inference.

## CONCLUSIONS

This investigation provides evidence of a significant dose-response relationship between serum cotinine concentrations and spine fracture risk in US adults. Our results indicate that interventions aimed at reducing tobacco smoke exposure, particularly through smoking cessation, could be relevant for strategies aimed at reducing the population burden of these fractures. Additionally, serum cotinine measurement shows promise as a potential biomarker for stratifying individuals according to fracture risk. These findings extend the existing evidence base and underscore the importance of future prospective studies to confirm these observations and elucidate the underlying mechanisms.

## Supplementary Material



## Data Availability

The data supporting this research are available from the authors on reasonable request. All the raw data required in this study can be extracted from the NHANES online website.
