# Supplementary material for: Tobacco smoke exposure and spine fracture risk in US adults: A cross-sectional analysis of the National Health and Nutrition Examination Survey 1999–2010, 2013–2014, 2017–2020
Source: Tob Induc Dis. 2026 Jun 8;24:10.18332/tid/217959. doi: 10.18332/tid/217959 (PMC13248791; doi:10.18332/tid/217959)
Supplement: Supplementary file 1 [file TID-24-83-s1.pdf]

Supplementary table 1: Logistic univariable regression analysis the Association between covariates and spine fracture using NHANES data, United States,1999-2010, 2013-2014, 2017-2020 (N = 31,124)

**Variable**	**OR_95CI**	***P*-value**
Age (Years)	1.02 (1.01~1.02)	<0.001
Gender:Female vs male	0.69 (0.6~0.8)	<0.001
Race		
Non-Hispanic White	1(reference)	
Non-Hispanic Black	0.3 (0.23~0.39)	<0.001
Mexican American	0.53 (0.43~0.67)	<0.001
Other	0.73 (0.58~0.9)	0.004
Educational level(Years)		
<9	1(reference)	
9-12	1.16 (0.9~1.5)	0.258
>12	1.15 (0.89~1.47)	0.287
Marital Status		
Married or living with partners	1(reference)	
Living alone	0.96 (0.83~1.12)	0.635
PIR		
Low income	1(reference)	
Medium income	0.88 (0.74~1.05)	0.152
High income	0.81 (0.67~0.98)	0.026
TC(mmol/L)	1.03 (0.96~1.09)	0.434
HDL-C(mmol/L)	0.74 (0.62~0.89)	0.001
Serum calcium(mg/dl)	1.02 (0.85~1.23)	0.849
Serum phosphorus(mg/dl)	0.9 (0.79~1.03)	0.113
ALT(U/L)	1 (1~1)	0.12
AST(U/L)	1 (1~1)	0.054
BUN(mg/dl)	1.01 (1~1.02)	0.033
UA(mg/dl)	1.06 (1.01~1.12)	0.013
WC(cm)	1.01 (1.01~1.02)	<0.001
BMI(kg/m2)	1.05 (1.05~1.06)	<0.001
Alcohol use:Yes vs No	1.68 (1.39~2.03)	<0.001
Hypertension:Yes vs No	1.49 (1.29~1.72)	<0.001
Diabetes:Yes vs No	1.49 (1.24~1.8)	<0.001
CHD:Yes vs No	1.75 (1.35~2.28)	<0.001
Stroke:Yes vs No	2.73 (2.14~3.49)	<0.001
Arthritis:Yes vs No	3 (2.59~3.48)	<0.001

Abbreviations: PIR, poverty to-income ratio. TC, total cholesterol. HDL-C, high-density lipoprotein cholesterol. ALT, alanine aminotransferase. AST, aspartate aminotransferase. BUN, blood urea nitrogen.UA, uric acid. WC, waist circumference. BMI, body mass index. CHD, coronary heart disease.

Supplementary table 2: Subgroup analysis of the association between tobacco smoke exposure and spine fracture using NHANES data, United States,1999-2010, 2013-2014, 2017-2020 (N=31,124)

Subgroup	N	Event (%)	OR (95%CI)	*P* for interaction
**Age**				
<55years	15,911	310 (1.9)	1.04 (1.01~1.08)	0.17
⩾55years	15,213	438 (2.9)	1.03 (1~1.06)	
**Gender**				
Male	15,271	433 (2.8)	1.05 (1.02~1.07)	0.152
Female	15,853	315 (2)	1.03 (1~1.07)	
**Race**				
Non-Hispanic White	15,403	495 (3.2)	1.05 (1.03~1.08)	0.393
Non-Hispanic Black	6,062	59 (1)	1.03 (0.96~1.11)	
Mexican American	5,452	95 (1.7)	0.99 (0.93~1.06)	
Other	4,207	99 (2.4)	1.04 (0.98~1.1)	
**Marital status**				
Married or living with a partner	19,381	472 (2.4)	1.06 (1.03~1.09)	0.077
Living alone	11,743	276 (2.4)	1.01 (0.98~1.05)	
**PIR**				
<1.3	8,695	234 (2.7)	1.04 (1~1.07)	0.693
1.3-3.5	12,040	286 (2.4)	1.04 (1~1.07)	
⩾3.5	10,389	228 (2.2)	1.06 (1.02~1.1)	
**Alcohol use**				
No	8,063	130 (1.6)	1.08 (1.03~1.14)	0.055
Yes	23,061	618 (2.7)	1.04 (1.01~1.06)	
**Hypertension**				
No	19,292	393 (2)	1.05 (1.02~1.08)	0.935
Yes	11,832	355 (3)	1.03 (1~1.07)	
**Diabetes**				
No	26,987	610 (2.3)	1.05 (1.02~1.07)	0.225
Yes	4,137	138 (3.3)	1.02 (0.97~1.07)	
**CHD**				
No	29,545	685 (2.3)	1.05 (1.02~1.07)	0.342
Yes	1,579	63 (4)	1 (0.92~1.08)	
**Stroke**				
No	29,840	672 (2.3)	1.05 (1.02~1.07)	0.462
Yes	1,284	76 (5.9)	1 (0.93~1.07)	
**Arthritis**				
No	21,587	327 (1.5)	1.04 (1.01~1.07)	0.234
Yes	9,537	421 (4.4)	1.03 (1.01~1.06)	

Abbreviations: LN, natural logarithm. OR, odds ratio. CI, confidence interval. PIR, poverty to-income ratio. CHD, coronary heart disease.

© **2026 Zhang R. et al.**
